# The impact of the COVID-19 pandemic on the provision of instrumental help by older people across Europe

**DOI:** 10.3389/fsoc.2022.1007107

**Published:** 2022-11-09

**Authors:** Michael Bergmann, Magdalena Viktoria Hecher, Elena Sommer

**Affiliations:** ^1^Munich Center for the Economics of Aging (MEA), Max Planck Institute for Social Law and Social Policy, Munich, Germany; ^2^Chair for the Economics of Aging, Technical University of Munich, Munich, Germany

**Keywords:** SHARE, COVID-19 pandemic, social cohesion, instrumental help, informal help, intergenerational exchange, solidarity

## Abstract

The outbreak of the COVID-19 pandemic in early 2020 introduced new challenges to social cohesion across Europe. Epidemiological control measures instituted in almost all European countries have impacted the possibility to provide help to others. In addition, individual characteristics contributed to whether individuals were able and willing to provide help to or receive help from others. Against this background, we focus on how private support networks of individuals aged 50 years and older across Europe were directly or indirectly affected by the COVID-19 pandemic. The focus of the paper is on the supply side. While the older population has been mainly perceived as recipients of instrumental help in the COVID-19 pandemic, the paper examines the patterns of providing instrumental help to others by the older generations and their changes during the pandemic. Has the provision of instrumental help increased or decreased in the course of the COVID-19 crisis? Have the groups of recipients changed during the pandemic? What were key determinants for helping others in 2021 as compared to the first phase of the pandemic 1 year before? And how did this differ across countries with different degrees of affectedness by COVID-19? To answer these questions, we analyzed representative data from the Survey of Health, Aging and Retirement in Europe (SHARE) and, in particular, the two waves of the SHARE Corona Survey, fielded in 27 European countries and Israel in 2020 and 2021. Results based on data from more than 45,000 respondents aged 50+ showed that help from children to parents has strongly increased in the first phase of the pandemic, while the opposite (parents helping their children) has decreased–especially in countries that have been hit hardest by the pandemic in 2020. This changed with the continuing crisis. Instrumental help provided to non-kin that was common in Western Europe in the first phase of the pandemic, yielding an optimistic view of increasing solidarity after the outbreak of COVID-19, strongly decreased 1 year later. Our findings provide a contribution to comparative research on micro- and macro-determinants that are crucial for the understanding of intergenerational support in times of crisis.

## Introduction

The outbreak of the COVID-19 pandemic in early 2020 introduced new challenges to social cohesion across Europe. On the one hand, people had the perception of a widespread willingness to help each other and saw “a lot of the best in humanity” during the lockdown time (Schneiders et al., 2022: 7). But on the other hand, there was also a “fear of being in contact with other people […] and feelings of distrust, judgement and tension within […] communities” (Schneiders et al., 2022: 7). A British study found that despite the general perception that people were willing to help each other, taken together, there was a decline in helping each other compared to the pre-pandemic situation (Borkowska and Laurence, 2021). However, so far there is a lack of research analyzing determinants affecting the provision of informal help across Europe during the COVID-19 pandemic.

The COVID-19 pandemic led to the implementation of various national policies and measures aiming at halting the spread of the virus through the reduction of in-person contacts. Such unprecedented measures faced a challenge for social cohesion in general, and exchange of instrumental help in particular, as it often requires in-person contact. At the same time, the exchange of informal help (e.g., help with groceries or house repairs) became even more important in the light of pandemic-related reduced availability of formal care service providers and social isolation especially for individuals under quarantine as well as high-risk groups such as older people. Therefore, the research on social cohesion and exchange of informal help in times of the still ongoing pandemic is highly relevant, especially research focusing on vulnerable social groups. The contribution of this paper is its focus on older people who are at highest risk of a severe course of the coronavirus disease and thus are highly affected by the pandemic.

In particular, the COVID-19 pandemic has challenged the solidarity between generations resulting in intergenerational tensions due to changing mutual expectations and obligations of older and younger people (Ayalon, 2020; Ellerich-Groppe et al., 2020; Ayalon et al., 2021; Stok et al., 2021). The political discourse in Western countries was mostly dominated by encouraging age separation and self-isolation of older people. Older generations were largely perceived as a homogeneous and fragile group defined solely by age that is in need of help in the times of a major health crisis and, in the beginning of the COVID-19 pandemic, younger generations were asked to show solidarity with older generations by adopting social distancing as a preventative measure (Graefe et al., 2020; Meisner, 2021; Stok et al., 2021). That upward intergenerational solidarity in the beginning of the COVID-19 pandemic has shifted to a call for downward solidarity from older to younger generations (e.g., appeal to older population to stay at home to enable lessening of protective measures for young people) in order to reduce detrimental effects of the pandemic for young people in later stages of the pandemic (Stok et al., 2021). Increased ageism, ignoring the heterogeneity of the older population when characterizing older people as the main recipients of support during the COVID-19 pandemic and underestimating the intergenerational support provided by older people (e.g., with childcare) have been recently criticized by several scholars (e.g., Ayalon et al., 2021; Vervaecke and Meisner, 2021). To quote Vervaecke and Meisner (2021: 163): “We must recognize that older adults in many instances and cultures are net providers (rather than receivers) of help and care through various roles, such as volunteers and unpaid caregivers of peers, spouses, and grandchildren.” In line with this criticism, in our study we focus on the agency and potentials of older people as providers of practical informal help to others in the times of the COVID-19 pandemic taking into consideration individual factors and the heterogeneity of the older population.

Since the beginning of the COVID-19 pandemic, researchers have been analyzing the impact of the pandemic on social cohesion and solidarity (e.g., the research initiative “Solidarity in times of a pandemic: What do people do, and why?”^1^). So far, research on social cohesion during the COVID-19 pandemic focused mainly on the United States and Great Britain (e.g., Stokes and Patterson, 2020; Borkowska and Laurence, 2021; Lalot et al., 2021; Jaspal and Breakwell, 2022; Schneiders et al., 2022). Current research on social cohesion in Germany shed light on the mental health perspective (e.g., Silveira et al., 2022), while an Austrian study investigated solidarity and social trust during the COVID-19 pandemic (Bodi-Fernandez et al., 2022). There are cross-national European studies as by Bergmann and Wagner (2021) and Tur-Sinai et al. (2021) discussing the development of care during the pandemic. While the focus of these studies is more on informal caregiving and care receiving, there is still a research gap with regard to intergenerational exchange of more common forms of (informal) help in a cross-national perspective [e.g., see Brandt et al. (2021) for Germany]. Our study aims at shedding light on this particular aspect, especially since it is closely connected to social cohesion and thus the positive effects of it (Berkman, 2000; Berger-Schmitt, 2002). The study provides a contribution to comparative research on micro- and macro-determinants that are crucial for the understanding of intergenerational support in times of health crisis. Little is known so far how usual patterns of intergenerational solidarity have changed in the context of a pandemic and restricted in-person contacts across Europe. The study takes into consideration country differences and explores cross-national variations that reflect country-specific developments of the pandemic as well as national pandemic-related policies and measures.

We use the representative cross-national data of the Survey of Health, Aging and Retirement in Europe (SHARE; Börsch-Supan et al., 2013), which is conducted in 27 European countries and Israel among households with individuals aged 50 years or older. In particular, this paper uses the data from the first and the second wave of the SHARE Corona Survey (SCS1 in 2020 and SCS2 in 2021) to explore changes in the provision of informal help in the course of the pandemic, taking into consideration heterogeneity of the givers and the cross-national context. The paper starts with a brief overview of the relevant conceptual and empirical background followed by a methodological overview. In the analytical part of the paper, we first explore regional differences with regard to the weighted prevalence of provided help in the first phase of the pandemic in summer 2020 compared to 1 year later. Afterwards, we analyze determinants of providing help and whether there are substantial differences between the first and the second wave of the SHARE Corona Survey, i.e., between 2020 and 2021. The paper concludes with a discussion of the main findings, study limitations and suggestions for future research.

## Materials and methods

### Previous research and hypotheses

#### Provision of instrumental help and the COVID-19 pandemic

Social cohesion usually refers to the interactions among members of a society which are characterized by a set of attitudes and norms including trust, a sense of belonging and the willingness to participate and help as well as their behavioral manifestations (Berkman, 2000). It is associated with a decrease of inequalities within a society and considered as a source of wealth and economic growth and health (Berkman, 2000; Berger-Schmitt, 2002). This applies also to the COVID-19 pandemic. A recent study by Silveira et al. (2022) that investigated the correlation of psychological indicators of vulnerability, resilience and social cohesion, supports this assumption. It found, that during the German lockdown respondents with higher levels of social cohesion showed a better mental health recovery in overcoming the multiple challenges of the crisis (Silveira et al., 2022). Therefore, also beyond the pandemic, social cohesion is promoted through social policies and is desired to be fostered by regional redistribution and active citizens (Easterly et al., 2006). Social cohesion is a broad multidimensional concept with varying definitions depending on the focus of a certain conceptualization and operationalization (Berkman, 2000). In our study, we focus on a particular behavioral aspect of social cohesion, namely on the willingness to help each other in form of informal instrumental help.

Given the fast development of the COVID-19 pandemic after its outbreak in Europe in early 2020, especially informal and thus more flexible help provided by active citizens can be of great importance for a functioning society. Older people, in general, are at higher risk of social isolation and loneliness and thus poor mental health as well as physical health conditions. Therefore, their inclusion and participation in a strong social network plays a significant role in the onset of depression and anxiety in the population as a whole. During the COVID-19 pandemic, older people became an even more vulnerable group facing higher risk of severe symptoms of COVID-19 and being affected by restricted access to formal care (Bergmann and Wagner, 2021). In light of this development, intergenerational solidarity has become particularly important. At the same time, the pandemic-related policies (e.g., in-person contact restrictions and physical distancing) and the risk of infection as well as changing expectations and obligations across generations presented new challenges for intergenerational solidarity.

In general, intergenerational solidarity that distinguishes structural, associative, affectual, consensual, normative and functional solidarity (Bengtson and Roberts, 1991) refers to social cohesion between generations including formal welfare support and exchange of emotional support as well as the exchange of financial transfers and instrumental help among family members and to others (e.g., help to obtain necessities like food and medications or emergency household repairs). In this context, solidarity means providing assistance when needed as part of bonding between different generations (Bengtson and Oyama, 2010). Previous research has demonstrated that formal intergenerational solidarity (e.g., welfare support, pensions, institutionalized care) flows mainly upward to older generations, whereas informal solidarity (e.g., transfers of private money and time) are usually directed downward from older generations to younger ones (Stok et al., 2021). Exchange of resources and assistance across generations is not only characterized by solidarity but also by conflict, as there is a need to (re)negotiate and balance the expectations and the flow directions of the resources exchange (Bengtson and Oyama, 2010). In the times of a major health crisis, there is more at stake than money and time, as the exchange of help is associated with additional burdens and costs (e.g., risk of getting infected, limitation of in-person contacts) and raises the question of fair allocation of burdens and benefits of integrational solidarity (Stok et al., 2021).

In our paper, we focus on the provision of informal instrumental help as one particular aspect of functional solidarity. We assume that this form of support is especially challenged by the COVID-19 pandemic and accompanying physical distancing measures as it usually requires in-person contacts, while other types of support (e.g., emotional or financial help) still can be provided without face-to-face contact. The likelihood of providing informal instrumental help depends on a mixture of individual attributes and macro factors. Generally, it is interpreted as an interplay of the needs of the potential recipients on the one side and resources as well as ability and willingness of the givers to provide certain type of help on the other side (Eggebeen, 1992; Vogel and Sommer, 2013). At least at the beginning of the pandemic, older people were presented in the general public discourse as a homogeneous fragile group in need of help, while individual attributes were not taken into consideration (Ayalon, 2020; Ellerich-Groppe et al., 2020; Graefe et al., 2020; Vervaecke and Meisner, 2021). Such rather paternalistic perspective can be seen as a form of “compassionate ageism” (Vervaecke and Meisner, 2021) which neglects the agency of older people to actively get engaged in the exchange of social support as providers of practical help. To shed light on the contribution of older people to the provision of instrumental help in European societies, our paper mainly focusses on providing rather than on receiving instrumental help. At the same time, we include reciprocity as an important factor for the provision of instrumental help and investigate various individual factors that might increase or decrease the likelihood of becoming a provider of instrumental help in later life during the COVID-19 pandemic.

During the pandemic, European countries were faced with completely new challenges regarding care provision for people aged 50+ (Bergmann and Wagner, 2021) and exchange of support between generations (Gilligan et al., 2020). Older people, especially those with poor health, were at high risk of experiencing a severe course of a coronavirus disease and to some extent were dependent on the help provided by others during the pandemic. Especially for older individuals who experienced restrictions to formal care and public support in the course of the pandemic intergenerational solidarity became a major resource for support during that time. Younger cohorts of a so-called “older population” (which we define here as people aged 50 years and older due to our sample), that are largely still in good health and occupationally active are expected to be providers of instrumental help during the COVID-19 pandemic. Especially in the beginning of the pandemic, younger generations were asked to show solidarity with older generations and to support them (Vervaecke and Meisner, 2021). Therefore, *a stronger provision of instrumental help can be expected in the beginning of the COVID-19 pandemic by younger cohorts of the older population (Hypothesis 1a)* to compensate for reduced access to formal care provision and to social networks outside the family due to contact restriction policies during the peaks of the pandemic.

At the same time, the adherence to lockdown restrictions and general practice of social and physical distancing to reduce the risk of infection, especially during the peaks of the pandemic, made it more challenging to provide support that requires personal interactions. The provision of help that requires personal contact had to be carefully evaluated upon the possible risk of infection vs. benefit of the received help. Especially the representatives of the middle generations were faced with competing demands and the double burden of providing support to their own children and older parents simultaneously (Gilligan et al., 2020; Stokes and Patterson, 2020). There was also a general shift in public debates calling for downward intergenerational solidarity in the later phases of the pandemic in order to reduce burdens associated with preventative measures for younger generations (Stok et al., 2021). Therefore, *less provision of instrumental help can be expected with the ongoing pandemic especially by younger cohorts of the older population (Hypothesis 1b)* if these were faced with an ongoing high (double) burden, leading to increasing difficulties in providing help the longer the pandemic and its accompanying restrictions continue. In addition, there was probably less need for help in the times of the “downtime” of the pandemic but also due to the vaccination of high-risk groups after the authorization of effective vaccines starting end of 2020.

#### Individual determinants regarding the exchange of instrumental help

Exchange of instrumental help is strongly associated with sociodemographic, economic, health-related and behavioral characteristics of givers and recipients like, for example, age, gender, education, income, health, social network and perceptions of reciprocity of exchange (Lowenstein and Daatland, 2006; Albertini et al., 2007; Litwin et al., 2008). Some older individuals tend to get involved in exchange of instrumental help to a higher degree than others do. To reflect this heterogeneity within the group of older individuals, our study analyzes crucial individual determinants of providing instrumental help.

With regard to socio-demographic and economic characteristics, previous research based on the cross-national SHARE data has shown that there is a general downward flow from the older to the younger generations for financial and practical assistance in European countries (Albertini et al., 2007; Litwin et al., 2008). Parents are more often the givers of help to children (even if these children are adults) than recipients. However, this only holds up to a certain age. For individuals aged 80 years and older, this pattern takes the opposite direction and this group, on average, becomes more often the net recipients of intergenerational exchange (Vogel, 2010). Furthermore, as older cohorts are more at risk to develop severe health problems in case of a COVID-19 infection, they are more in need of getting help. Several studies dealing with intergenerational exchange also have demonstrated that females are more often the givers of instrumental help than males (e.g., Steinbach, 2013). At the same time, men also receive less help from their grown-up children as compared to women (e.g., Brandt et al., 2009). A study conducted by Borkowska and Laurence (2021) demonstrates that in Great Britain less-educated individuals reported to experience less positive changes compared to pre-pandemic times with regard to social cohesion than individuals with higher education. In terms of rural-urban divide, there is no clear-cut direction in the literature: several studies on social cohesion and volunteering have demonstrated higher levels of both in rural areas (e.g., Fortuijn and van der Meer, 2006; Svendsen and Svendsen, 2016), although the differences seem to have decreased recently (e.g., Paarlberg et al., 2022). Intergenerational contacts, on the other hand, tend to be higher if parents are living in large urban areas as they are more likely to have children living nearby given that the younger generation prefers living in cities (Daatland, 2007). As multigenerational households are also less common in urban than in rural areas (Scherger et al., 2004), more within-household exchange of instrumental help can be expected in rural areas, while the provision of instrumental help outside the own household, which is the focus of our study, might be more common in urban areas. In addition, it has been shown that individuals with a migration background and low income were less likely to participate in community activities during the pandemic and experienced a larger decline in social cohesion (Jaspal and Breakwell, 2022). This fits well with findings of a positive association between being employed and providing modest amounts of extra-resident support as having the financial resources might facilitate the provision of informal care and help (Arber and Ginn, 1992, 1995). Based on this previous research, we expect that *being older, male, less educated and having a migration background are associated with lower provision of instrumental help during the COVID-19 pandemic, while living in urban areas, having a paid work and a high income are associated with higher provision of instrumental help (Hypothesis 2)*.

Regarding health-related outcomes, individuals with long-term health conditions were found to have a less strong social network in general and tended to engage less in social activities during the COVID-19 pandemic (Jaspal and Breakwell, 2022). Further, an exposure to COVID-19 might also affect the willingness and ability to provide instrumental help to others (see the argumentation in Bergmann and Wagner, 2021 regarding the provision of care). Knowing people in their own social circles who have been infected with the coronavirus might increase the likelihood of providing instrumental help, simply as there is need for it (e.g., helping with groceries for persons in quarantine). Individuals affected by COVID-19 themselves, on opposite, are possibly less likely to provide instrumental help to others, especially in case of severe or long-term symptoms of COVID-19. Therefore, we expect that *knowing people exposed to COVID-19 in their own social circles is positively associated with the provision of instrumental help, while being self-exposed to COVID-19 as well as experiencing poor health in general is negatively correlated with the provision of instrumental help during the pandemic (Hypothesis 3)*.

In addition, also behavioral characteristics are linked to the provision of help. People with a higher number of (in-person) social contacts seem to have more occasions to provide instrumental help to others. Vergauwen et al. (2022) found that, despite stringent contact policies during the COVID-19 pandemic, older adults were generally not likely to experience a decrease in contacts and assumed that increased support (including digital contacts) for parents might explain this effect. Further, reciprocity is seen to play an important role in the exchange of social support. Reciprocal intergenerational exchange is related to better psychological well-being (Silverstein and Bengtson, 1991; Lowenstein et al., 2008). Various studies have shown that persons who receive help are more likely to provide help in return (Pruitt, 1968; Wilke and Lanzetta, 1970; Kahn and Tice, 1973). Reciprocity of intergenerational solidarity became especially important in the later phase of the pandemic, when younger generations appealed to older generations for their support to balance competing needs of different generations (Stok et al., 2021). Against the background of these considerations, we expect that *during the COVID-19 pandemic, having frequent social contacts and being a receiver of instrumental help are associated with a higher provision of instrumental help (Hypothesis 4)*.

#### Cross-national differences

The ability and willingness of providing instrumental help is not only dependent on individual factors, some of which were mentioned above, but is also linked to macro factors. European countries introduced different policies as a response to the pandemic and the pandemic-related epidemiological control measures varied to a high extent with regard to their level of stringency across Europe (Hale et al., 2021). In addition, despite the fact that all European countries were affected by the pandemic, they were affected by it to a different extent and at different times. While some studies found that stricter policies (e.g., strict distancing and limitation of personal contacts as well as stay-at-home orders) were associated with less provision of formal care services (e.g., Benzeval et al., 2020; Eggert et al., 2020; Moss, 2020; Wolf-Ostermann et al., 2020), several studies show that more informal contact and support was actually provided to compensate for the greater demand by older people (Arpino et al., 2020; Bergmann and Wagner, 2021; Vergauwen et al., 2022). We therefore expect *more provision of instrumental help during the COVID-19 pandemic in countries with more strict pandemic-related policies and measures (Hypothesis 5)*.

### Methodology

#### Data and sample

In our analyses, we use data from the regular SHARE waves (Börsch-Supan, 2022a,b,c,d,e,f,g,j) and from the first and the second SHARE Corona Survey (Börsch-Supan, 2022i,l). SHARE is based on full probability samples (Bergmann et al., 2019, 2021, 2022b), providing internationally comparable representative data for the 50+ population. Both the methodological rigor and the ex-ante cross-national harmonization of SHARE are particularly suitable to investigate the effects of a global crisis like the COVID-19 pandemic. The regular SHARE is a longitudinal survey fielded every 2 years *via* face-to-face interviews with individuals aged 50 years and older and their partners living in the same household. In our analyses, we use data from the regular SHARE waves for the information on stable respondent characteristics, such as education level and health conditions.

The SHARE Corona Surveys were introduced as telephone interviews in order to enable timely data collection on pandemic-related topics. Longitudinal SHARE respondents were invited to participate in the first SHARE Corona Survey that was fielded in June and July 2020. The second SHARE Corona Survey re-interviewed respondents from the first survey in summer 2021, enabling the examination of changes between the start of the pandemic and the situation about 1 year later. The average response rate based on eligible respondents participating in the first SHARE Corona Survey was 79 percent. In the second SHARE Corona Survey, an average retention rate (excl. recovery of respondents) of 86 percent was achieved. To avoid selectivity, our analyses are based on 47,495 respondents who participated in both SHARE Corona Surveys. Among other pandemic-relevant content, both SHARE Corona Survey questionnaires contain a section on social networks that includes questions about providing and receiving instrumental help, which build the basis for our analyses. We further included country-specific information on epidemiological control measures using data from the Oxford COVID-19 Government Response Tracker (OxCGRT; Hale et al., 2021) that are available on a daily basis.

#### Measures

To examine the factors related to the provision and the receipt of instrumental help during the pandemic as well as the changes in exchanging instrumental help in the course of the pandemic, we used the following variables from the first and second SHARE Corona Surveys.

First SHARE Corona Survey (SCS1 in 2020):

1. Since the outbreak of Corona, did you help others outside your home to obtain necessities, e.g., food, medications or emergency household repairs? Yes/No.
2. Compared to before the outbreak of Corona, how often did you help the following people (Own children; Own parents, Other relatives; Other non-relatives like neighbors, friends or colleagues) from outside your home to obtain necessities: less often, about the same, or more often?

Second SHARE Corona Survey (SCS2 in 2021):

1. Since the outbreak of Corona, have you helped the following people (Own children; Own parents; Other relatives; Other non-relatives like neighbors, friends or colleagues) outside your home to obtain necessities, e.g., food, medications, or emergency household repairs? Please answer yes or no to each category.
2. Compared to the first wave of the pandemic, how often did you help (Own children; Own parents; Other relatives; Other non-relatives like neighbors, friends or colleagues) to obtain necessities in the last 3 months, e.g., food, medications, or emergency household repairs? Less often, about the same, or more often?

The multivariate analysis controls for a number of correlates known from previous research on intergenerational exchange mentioned above. As socio-demographic and economic characteristics, we used *respondents' age* at the respective interview in 2020 (SCS1) and 2021 (SCS2) to form three age groups (50–64 years, 65–79 years, 80 years and older) and *respondents' sex* (0: male, 1: female) from the coverscreen data of the regular SHARE interview. Further, we coded the *level of education* attained based on the Internal Standard Classification of Education 1997 (ISCED-97) by using information from the respondents' baseline interview. Respondents were then grouped into two categories: primary education (ISCED-97 score: 0–2), secondary and post-secondary education (ISCED-97 score: 3–6). We further used information on the *respondents' country of birth* from the regular SHARE interview to determine whether they were born abroad or not as well as the *type of living area* (0: rural area, 1: urban area like a large town or big city). The latter information was updated during the second SHARE Corona Survey in case of moving. We further included a measure related to whether respondents were *employed* (including self-employment) or not at the time when Corona broke out (SCS1)/at the time of the interview (SCS2). In addition, we measured *respondents' economic status* by a question that asked the degree to which they were able to make ends meet (0: with great/some difficulty, 1: fairly easily/easily) since the outbreak of Corona (SCS1)/since the last interview (SCS2)^2^.

To control for *respondents' health*, we used the reversed 5-point scale on their self-rated health (0: poor, 1: fair, 2: good, 3: very good, 4: excellent) at the time of the respective SHARE Corona Survey and collapsed the categories poor and fair as well as good, very good and excellent to build a dichotomous indicator. In addition, we included a measure that indicates whether respondents were directly *affected by COVID-19* (self-exposure) by using a set of questions on (a) having experienced symptoms, (b) having been tested for COVID-19 and (c) having been hospitalized. To determine whether someone close to the respondent was affected (social exposure) by COVID-19, we used information on symptoms, tests, hospitalization and deaths due to COVID-19 with regard to the respondent's spouse/partner, parent, child, other household member, other relative outside the household, and neighbors, friend or colleague.

As behavioral measures, we used the *contact frequency* of respondents and summed up the frequency of face-to-face contacts (i.e., 4: daily, 3: several times a week, 2: about once a week, 1: less often, 0: never) with people from outside the household (i.e., own children, own parents, other relatives and other non-relatives like neighbors, friends, or colleagues). Based on this metric indicator, we applied a median-split to separate respondents with lower/higher than median contact frequency since the outbreak of Corona (SCS1)/during the last 3 months (SCS2). To measure *reciprocity of instrumental help*, we used respondents' answers on the question whether they were helped by others from outside of home to obtain necessities, e.g., food, medications or emergency household repairs or not since the outbreak of Corona. To shed light on the heterogeneity across respondents of different age groups and possible consequences thereof during the different phases of the pandemic, we included an interaction of receiving instrumental help with age (< 65 vs. ≥65 years).

Finally, we used the so-called *stringency index* from the Oxford COVID-19 Government Response Tracker (OxCGRT; Hale et al., 2021) to assess differences in national policy responses toward the pandemic. The index records the strictness of “lockdown style” policies, which primarily restrict people's behavior and in particular in-person contacts that are essential for the exchange of instrumental help. In particular, it aggregates policy responses about school and workplace closings, canceling of public events, restrictions on gatherings, closure of public transports, stay at home requirements, restrictions on internal movement, international travel controls and public information campaigns. The stringency index is the average of the mentioned policy indicators on a daily basis. It ranges from 0 to 100, with greater values indicating greater strictness. By matching the Oxford data to the SHARE Corona Survey data *via* the specific interview date of all respondents (Börsch-Supan, 2022h,k) we were able to match precisely the country-specific context information on the pandemic to the respondents' answers on the day of the interview. By this, we could use the full variation inherent in the data to improve our model estimations. We followed the operationalization by Bassoli et al. (2021) and summed up, for each country, all daily values of the stringency index since the 1^st^ of January 2020 until the respondent's individual interview date. Afterwards, we divided this value by the total number of days elapsed between January 1, 2020 and the interview date. As a result, countries that implemented lockdown policies later have a lower index. Further, if two countries had the same start date of lockdown policies, but different intensity, the country with stricter policies will have a higher stringency index value for the respective respondent.

#### Statistical analyses

To address our research questions, we first descriptively explored regional differences regarding the overall prevalence of providing instrumental help during the pandemic (1) as well as differentiated by type of relationship (2), considering the specific age structure of our sample. Afterwards, we investigated key determinants that were crucial for helping others during the first phase of the pandemic in 2020 compared to 1 year later to analyze substantial differences. For this, we used multivariate logistic models including a large set of individual respondent characteristics, such as respondents' age, sex, level of education, migration background, area of living (rural vs. urban), whether they were (self-) employed before the pandemic and subjective economic status. Furthermore, we analyzed respondents' self-rated health, their affectedness by COVID-19, their frequency of in-person contacts and whether they received help from others or not, which are closely linked to the provision of instrumental help. Finally, we included COVID-19-related policy measures (strictness and lengths of containing policies; linear and quadratic) at the country level, which have been transferred to the individual level by matching the stringency index to the actual date of the respondents' interview^3^. Moreover, we included country dummies to control for any additional regional differences. All variables were standardized with regard to the overall sample mean. Analyses were performed using Stata 14.1 based on robust standard errors and with calibrated cross-sectional weights as provided by the SHARE Coordination team.

## Results

### Prevalence of providing instrumental help across Europe

**Figure 1:**
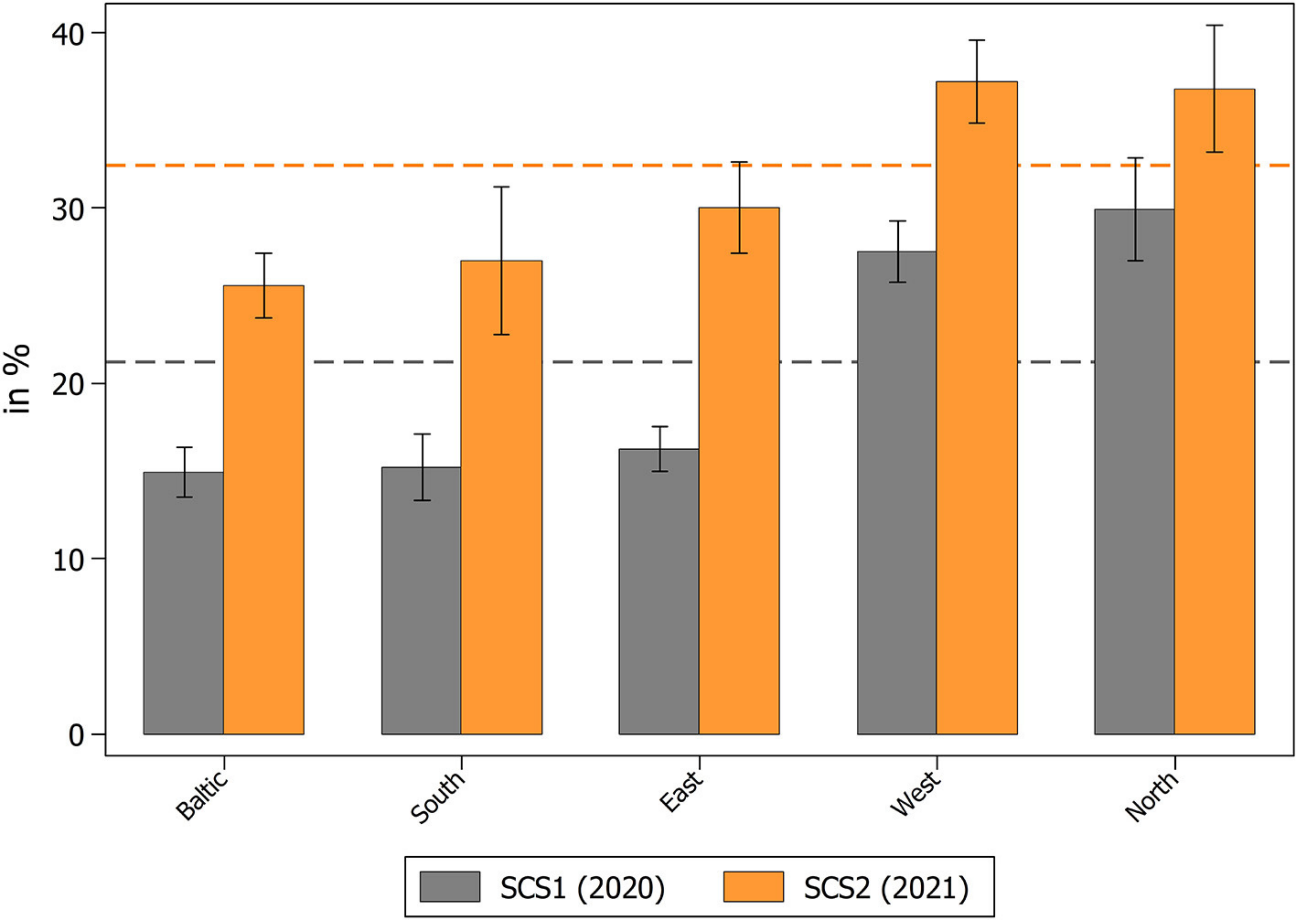
Percent of respondents providing help to others outside their own household since the outbreak of the pandemic. Data: SHARE Wave 8 COVID-19 Survey 1 and SHARE Wave 9 COVID-19 Survey 2, Release 8.0.0 (*n* = 47,495, respectively; weighted) with 95% confidence intervals.

We started our analyses with reporting the overall prevalence of providing instrumental help by individuals aged 50+ across Europe during the different phases of the pandemic. While the 2020 survey found that, on average, 21.2% (*n* = 7,452) of all respondents gave help to others outside the own household since the outbreak of the pandemic, the prevalence increased in the 2021 survey by more than ten percentage points to 32.4% (*n* = 11,864). Regional variation showed the strongest relative increase in Southern Europe (Croatia, Cyprus, Greece, Israel, Italy, Malta, Portugal, Slovenia, Spain) and Eastern Europe (Bulgaria, Czech Republic, Hungary, Poland, Romania, Slovakia). However, the increase was also considerable in the Baltic States (Estonia, Latvia, Lithuania) and in Western European countries (Austria, Belgium, France, Germany, Luxembourg, Netherlands, Switzerland). The smallest relative increase was found in Northern Europe (Denmark, Finland, Sweden; see Figure 1). When further investigating the effect of age on the provision of instrumental help (see Supplementary Table A1) it came as no surprise that the absolute level of providing help was much higher for younger respondents between 50 and 64 years (29.0% in SCS1 and 42.3% in SCS2) compared to older respondents aged 65 years and above (12.6% in SCS1 and 22.4 in SCS2).

There are two interpretations for this general pattern: First, due to the vaccination campaign, which started end of 2020 in most European countries and picked up speed in spring 2021 (see European Center for Disease Prevention and Control, ECDC), restrictions could be relaxed and social contact as well as support in general was possible again easier. Also, there was possibly less fear of suffering from severe COVID-19 symptoms for vaccinated individuals. In addition, in the course of the pandemic European countries introduced different policies to provide public support to their population which were not available in the beginning. Previous studies showed that European countries, in which families were relieved by welfare support provided by the state, were more stimulated to engage in the provision of informal intergenerational support as complementary help (“crowding-in;” Künemund and Rein, 1999; Silverstein et al., 2020). In addition, while in the early phase of the COVID-19 pandemic primarily the older population was addressed as being the group in need of help, in the course of the pandemic there was a call for a shift from upward to downward intergenerational solidarity and an appeal for the reciprocity of intergenerational exchange as younger generations were presented as those carrying the double burden of the pandemic (Ellerich-Groppe et al., 2020, 2021). Therefore, it seems plausible that especially older cohorts started engaging more in the provision of instrumental help in the later phase of the COVID-19 pandemic. This can be seen when looking at the relative increase between 2020 and 2021, which was stronger for older people aged 65+ (+78%) than for younger people between 50 and 64 years (+46%). A second, methodological explanation is based on the reference point (“since the outbreak of the pandemic”) that was used in both questionnaires. Respondents in the second SHARE Corona Survey hence simply had more time and opportunities to help others due to the longer reference period between the outbreak of the pandemic and the respective interview. It is thus likely that the increase of instrumental help during the pandemic that is evident from Figure 1 is an overestimation due to the questionnaire design. However, we are able to test this assumption based on a different question focusing on actual changes in the provision of help in the following section.

### Changes in the provision of instrumental help since the outbreak of and during the COVID-19 pandemic

**Figure 2:**
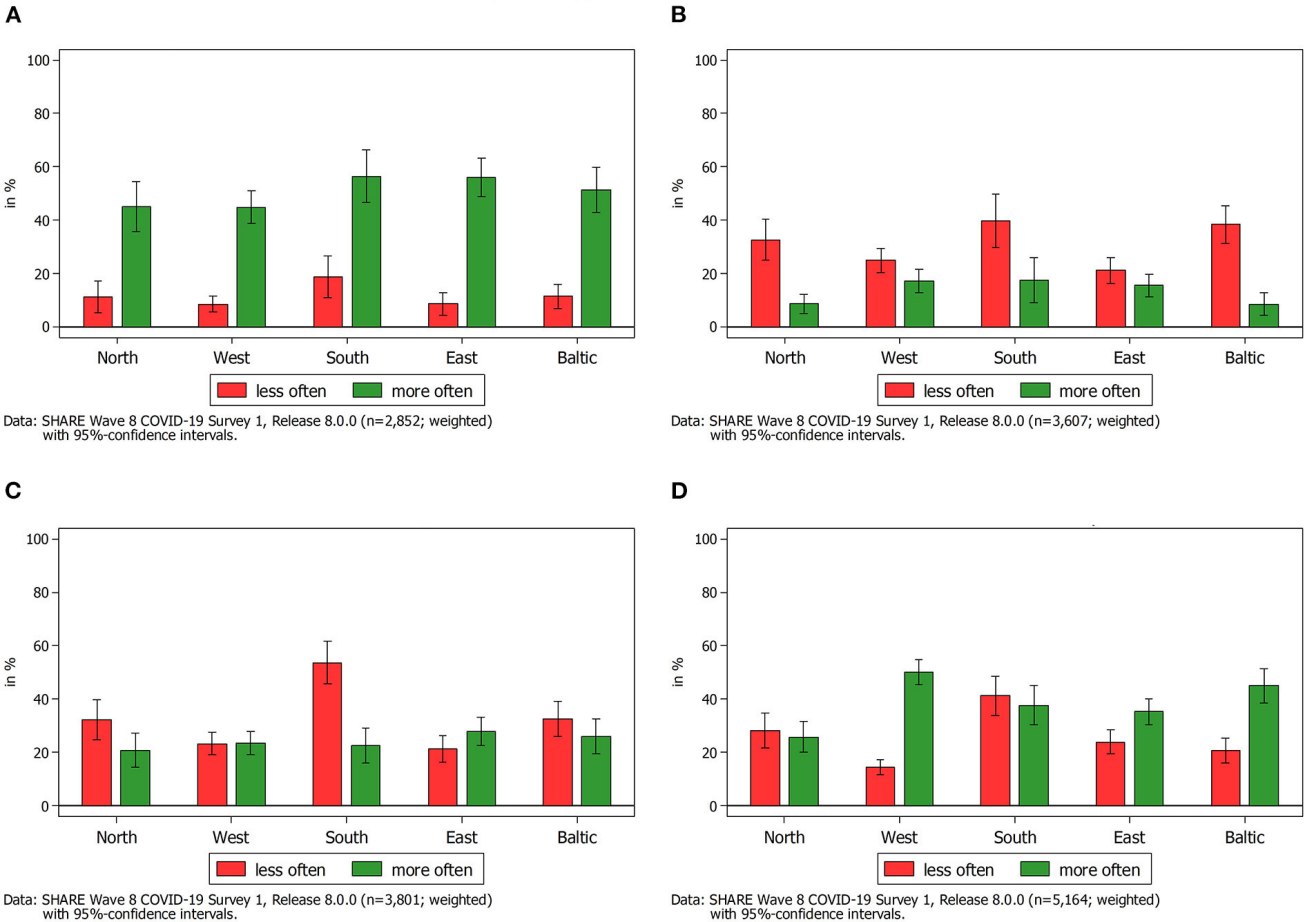
Change in frequency of providing help to someone outside the own household since the outbreak of the pandemic in 2020 by type of relationship. **(A)** Help provided to parents outside the household since the outbreak of the pandemic. **(B)** Help provided to children outside the household since the outbreak of the pandemic. **(C)** Help provided to other relatives outside the household since the outbreak of the pandemic. **(D)** Help provided to other non-kin outside the household since the outbreak of the pandemic.

**Figure 3:**
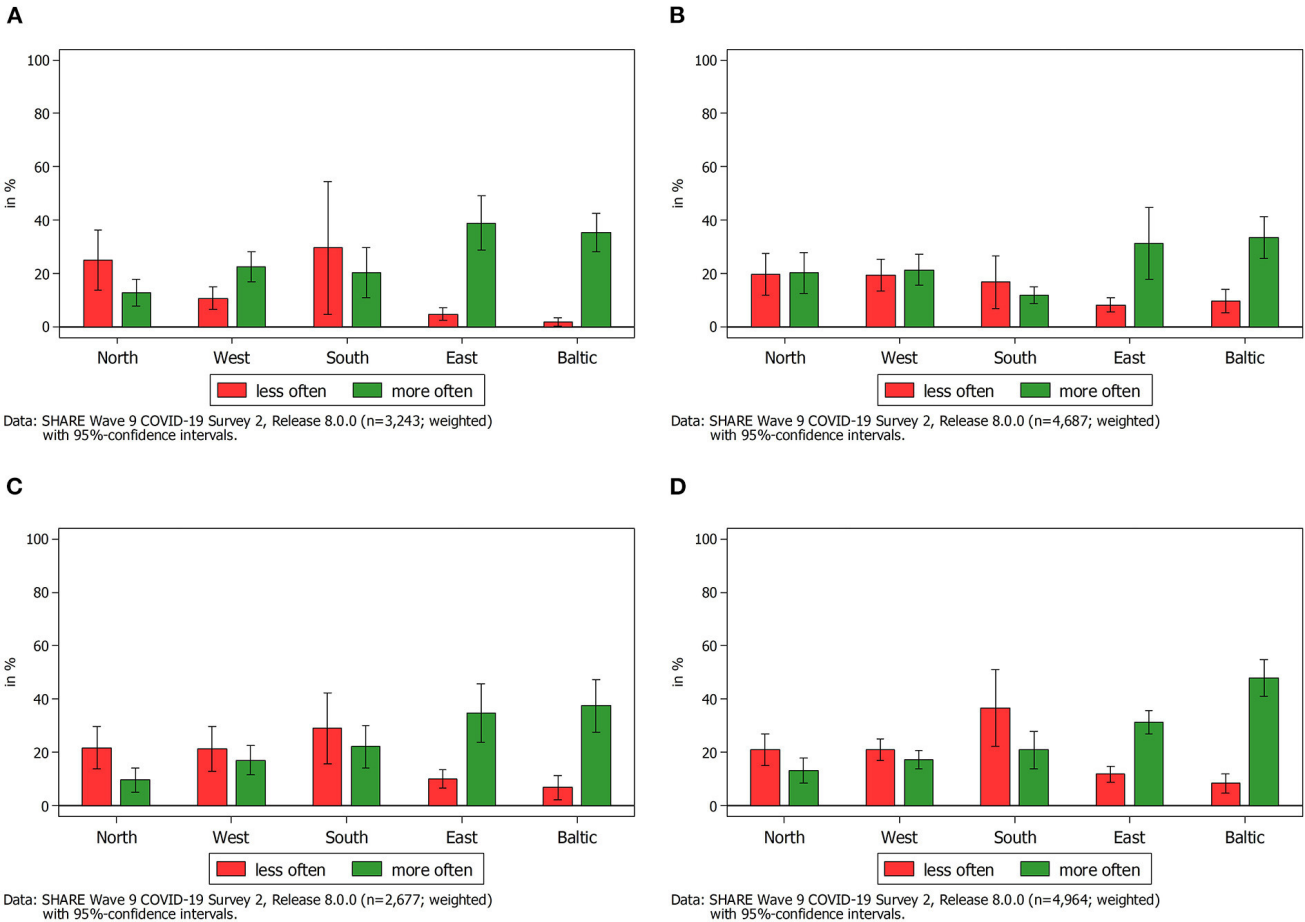
Change in frequency of providing help to someone outside the own household compared to the first wave of the pandemic by type of relationship. **(A)** Help provided to parents outside the household compared to the first wave of the pandemic. **(B)** Help provided to children outside the household compared to the first wave of the pandemic. **(C)** Help provided to other relatives outside the household compared to the first wave of the pandemic. **(D)** Help provided to other non-kin outside the household compared to the first wave of the pandemic.

In addition to the overall prevalence of providing instrumental help across Europe, we further analyzed changes thereof by different types of relationship (see Figures 2, 3). By this, we could investigate whether respondents aged 50 years and above reported an increase or a decrease in their provision of instrumental help to others since the outbreak of COVID-19 (SCS1 in 2020) as well as compared to the first wave of the pandemic (SCS2 in 2021). Moreover, the differentiation between children and parents as providers or receivers of instrumental help allowed us to analyze age-related differences in a straightforward way as respondents in SHARE providing help to their parents (including those simultaneously providing help to their children) are usually younger than respondents providing help to their (adult) children but not to their parents.

Against this background, we first looked at the reported changes in the first SHARE Corona Survey regarding the provision of instrumental help since the outbreak of the COVID-19 pandemic as compared to the time before the outbreak. Most striking in this respect was the large increase in providing help to parents in summer 2020 as compared to the pre-pandemic time, which is visible in the upper left graph of Figure 2A. This increase was rather consistent across Europe and confirmed previous findings with regard to personal care (Bergmann and Wagner, 2021). Between 45 percent (Western Europe) and 56 percent (Southern Europe) of all respondents, who provided any sort of instrumental help, declared that they had increased the provision of help to their parents since the outbreak of the pandemic. That is, on average, more than every second respondent reported an increase. In contrast, only between 8 percent (Western Europe) and 18 percent (Southern Europe) indicated that they had decreased the help given to their parents. The rest, on average about 38 percent, had neither increased nor decreased the provision of help to parents since the outbreak of the pandemic. This finding supported previous studies showing that parents aged 80 years or above are usually the receivers of instrumental help rather than the givers (Vogel, 2010). Given that SHARE respondents are 50 years or above, their parents are often older than 80 years and thus belong to the vulnerable group strongly affected by the COVID-19 pandemic and possibly need more support from family members than in pre-pandemic times.

The picture considerably changed when looking at the other subgraphs in Figure 2. With respect to parents providing help to their children outside their own household in 2020 (Figure 2B), nearly one third of all respondents, independent of their age, declaring provision of instrumental help since the outbreak of the pandemic reported a decrease in helping their children. In contrast, only every sixth respondent reported an increase in the provision of instrumental help. Thus, with the exception of the Western and Eastern European countries, decreases in the provision of help from parents to their children significantly outweighed the increases. These descriptive findings showed the opposite direction of providing intergenerational help in Europe demonstrated in previous studies. As mentioned before, there usually is a downward flow of help provision from parents below 80 years to their adult children (Albertini et al., 2007; Litwin et al., 2008). In the times of the COVID-19 pandemic there was, however, a general decrease in providing help to adult children. One possible explanation for this finding is that, based on the SHARE Corona Survey data, there was in general less in-person contact between SHARE respondents and their non-resident children in the first phase of the pandemic in 2020 as compared to 1 year later. In-person contacts, however, are often needed for the provision of instrumental help by parents to their adult children (e.g., looking after grandchildren). Thus, contact restrictions to contain the spread of the coronavirus as well as a higher risk for a severe course of the coronavirus disease especially for older people might have affected the provision of help to adult children outside the own household especially at the beginning of the COVID-19 pandemic when vaccines were still not available. At the same time, SHARE respondents, as shown above, have increased the provision of instrumental help to their parents–frequently involving less personal contact (e.g., grocery shopping)–and hence might have prioritized supporting them over the support of their children. In this respect, further analyses showed that when looking at respondents who reported helping their parents in the first phase of the pandemic, an even higher proportion of them declared that they provided less instrumental help to their children. This finding goes in line with the public discourse at the beginning of the COVID-19 pandemic that encouraged prioritizing support of older generations over support of younger generations.

With respect to other relatives and other non-kin, findings were more balanced (see Figures 2C,D). Notably exceptions were found in Southern Europe, where help given to other relatives strongly decreased during the first phase of the pandemic in 2020, and Western Europe (as well as to a lesser extent in the Baltic States and in Eastern Europe), where the reported increase in instrumental help provided to other non-kin was much stronger than the decrease. Whereas, this can be partly interpreted as indication for a positive development of social cohesion in the beginning of the COVID-19 pandemic, it is not possible to relate this finding to the intergenerational solidarity debate as the used categories “other relatives” and “non-kin” can include persons from different generations.

When comparing these results with the findings 1 year later (see Figure 3), several things are worth mentioning: First, although the overall proportion of SHARE respondents who reported to have provided instrumental help to others outside the own household since the outbreak of the COVID-19 pandemic has increased in 2021 (see Figure 1), the reported amount of giving help to someone more often was smaller as compared to 2020. Second, and even more striking, our findings revealed very different patterns regarding the type of relationship. While increases were rather comparable to decreases in Northern, Western and Southern Europe regarding instrumental help provided to parents, children, relatives and other non-kin, respondents in the Baltic States as well as in Eastern Europe reported a strong increase of providing instrumental help to others compared to the first phase of the pandemic in 2020. A possible explanation for this finding is that the rates of COVID-19 vaccinations were much lower in Eastern European countries and also in the Baltic States as compared to the rest of Europe (Bergmann et al., 2022a) and that at the same time infection rates were relatively high (Hale et al., 2021). Possibly, in those European countries with high vaccination rates the need for instrumental help decreased in general in summer 2021, resulting in a decrease of provided instrumental help.

### Determinants of providing instrumental help during the COVID-19 pandemic

**Figure 4:**
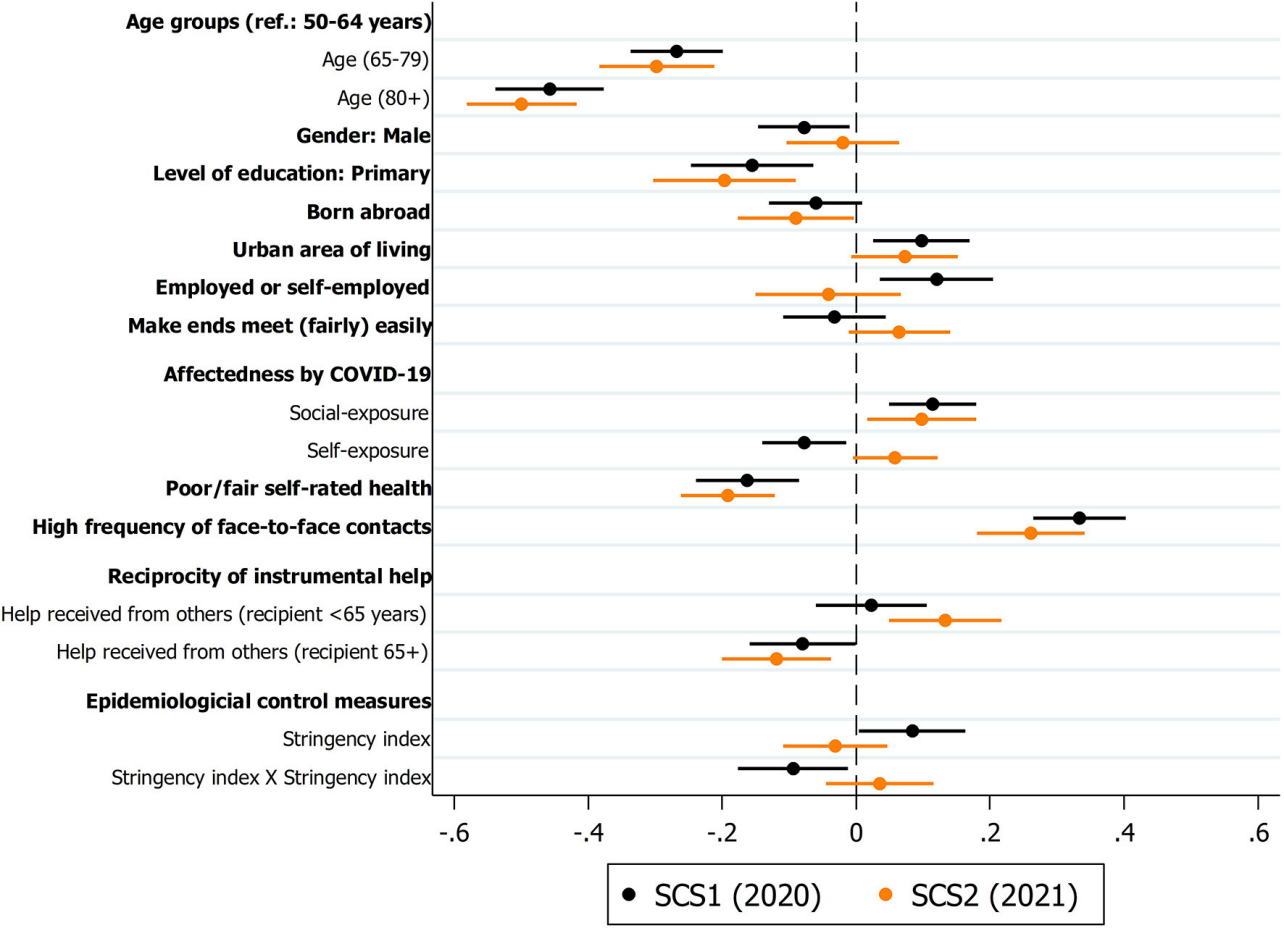
Multivariate logistic regression coefficients of respondent and country predictors on providing instrumental help to others. Data: SHARE Wave 8 COVID-19 Survey 1 and SHARE Wave 9 COVID-19 Survey 2, Release 8.0.0 (*n* = 42,918, respectively; weighted) with 95% confidence intervals.

Figure 4 graphically presents the coefficients of the respondent- and country-level predictors for the multivariate logistic regression model. The upper (lower) point estimate with 95% confidence intervals around represents the coefficients from the first (second) SHARE Corona Survey. Overall, the determinants explained about 11 (10) percent in the first (second) SHARE Corona Survey (the full models with all parameter estimates can be found in Supplementary Table A2). Substantially, we see that older respondents had a significantly lower probability of providing instrumental help since the outbreak of the pandemic. In addition, lower educated respondents with primary level of education had a significantly lower probability to provide instrumental help. In both cases, the differences between the first and the second SHARE Corona Survey in 2020 and 2021 were rather small and insignificant when using a z-test statistic to compare the differences between the coefficients (see last column in Supplementary Table A2). This was also the case for respondents' sex. However, while male respondents (compared to females) had a significantly lower probability of providing instrumental help in the first phase of the pandemic in 2020, this was not the case anymore 1 year later. In 2021, males provided only slightly (and at an insignificant level) less instrumental help to others outside the home than females. Respondents with a migration background provided less instrumental help both in in the first phase of the pandemic as well as 1 year later. However, while in 2021 the association was significant at the 95%-level, in 2020 it was only significant at the 90%-level. With regard to urban-rural differences a similar pattern as for gender was found: Only in the first phase of the pandemic living in an urban area had a significant positive effect on the probability to provide instrumental help. This effect decreased 1 year later in summer 2021, although still significant at the 90%-level. With respect to employment status, it could be seen that employed or self-employed respondents had a significant higher probability to provide instrumental help in the first phase of the pandemic in 2020. This association turned around 1 year later. In the second SHARE Corona Survey in summer 2021, (self-) employed respondents provided less instrumental help, although at an insignificant level. Nonetheless, the difference between the coefficients in 2020 and 2021 was significant. In contrast, respondents' subjective economic situation (“make ends meet”) did not exhibit a significant association with providing instrumental help.

Another rather strong effect was found with respect to respondents' self-rated health. Here, a worse physical health was associated with a significant lower probability to provide instrumental help during the course of the pandemic. As expected, affectedness by the coronavirus also played a role in explaining instrumental help: Respondents who knew someone in their social circles who was affected by COVID-19 had a higher probability to provide instrumental help in both 2020 and 2021. In addition, respondents who were directly affected themselves by a COVID-19 infection were found to provide less help – at least in 2020 and here also significant at the 95%-level. One year later and probably with more security by widespread vaccinations, the negative effect of self-exposure to COVID-19 disappeared completely and even turned positive, also leading to a significant difference between the coefficients in 2020 and 2021.

Regarding frequent contacts, we found a strong and positive correlation with providing instrumental help: Respondents, who reported high in-person contacts, in both surveys provided substantially more help to others since the outbreak of the pandemic. With respect to reciprocal behavior, there was evidence for a changed pattern in the course of the pandemic dependent on the age of the respondents: While a positive correlation between receiving and giving instrumental help was found for younger respondent (<65 years), the opposite was true with regard to older respondents (≥65 years). Interestingly, the positive correlation in the first phase of the pandemic for younger respondents was not statistically significant, indicating that providing help by this group was rather independent from receiving help during that time. This changed during the ongoing pandemic. Further, the found significant negative correlation for older people in 2020 and 2021 indicate that older people are more frequently the receivers (and not the providers) of instrumental help. Finally, there was some evidence that stricter control measures in the first phase of the pandemic were associated with providing more instrumental help, probably as a compensation of reduced formal help and care services. However, the negative correlation of the quadratic operationalization of the stringency index can be interpreted in the sense that very strict measures at the upper bound of the stringency index reduced the provision of help to some extent. This observation disappeared in 2021, meaning that continued control measures did not exhibit a significant effect on the provision of instrumental help anymore.

## Discussion

In terms of our hypotheses, the descriptive data analyses largely supported Hypothesis 1a (*a stronger provision of instrumental help can be expected in the beginning of the COVID-19 pandemic by younger cohorts of the older population)*. While this assumption seemed to hold for some specific groups of help recipients such as parents and non-relatives, the opposite was true for other groups of receivers like children and other relatives. In a study conducted by Silverstein et al. (2020) analyzing the so-called “sandwich generation” of older adults with alive parents and children, the authors demonstrated support for the “complementary giving” hypothesis for most European countries, i.e., generations were not competing for resources. Our findings, however, rather support the tendency of “competitive giving” with exhibiting more instrumental help for the older generation that probably was more in need of help–in particular at the beginning of the pandemic. In times of crisis such as the COVID-19 pandemic, the middle generation was faced with restricted access to public resources and formal care and, simultaneously, with competing demands for support from different potential recipients. Consequently, this group had to prioritize support toward those who needed it most: the older generation. This interpretation is also supported by other authors who identified older people due to their particular vulnerability as the main receivers of help in the beginning of the COVID-19 pandemic, while in later phases of the ongoing crisis also younger people have been recognized as addressees of intergenerational solidarity (e.g., Ellerich-Groppe et al., 2020). In this respect, it is interesting to see that the found relative increase of instrumental help between 2020 and 2021 was stronger with regard to older people above 65 years than for younger people between 50 and 64 years. Probably, the latter suffered more from an ongoing or even increasing (double) burden during the pandemic and thus might have had to restrict their support at some point. Nevertheless, further in-depth analyses are needed, considering the complex interplay of intergenerational exchange of different types of instrumental help (involving varying levels of burdens and risks when it comes to personal contact) that might also be subject to change over the individual life cycle.

Against this background and explicitly considering the age of respondents, Hypothesis 1b (*less provision of instrumental help can be expected with the ongoing pandemic especially by younger cohorts of the older population)* was also supported. This change in the course of the COVID-19 pandemic demonstrate the dynamics of the intergenerational solidarity in times of crisis as well as changing patterns and dependencies that have to be carefully considered when drawing conclusions. In this respect, our findings can be seen as a starting point that need to be supplemented by other studies adding further information on younger people below 50 years. However, what should be additionally noted based on our findings is that while in some parts of Europe there was a decrease of instrumental help provided to others 1 year after the start of the pandemic, in other parts (mainly Eastern Europe and the Baltic States) there was, on contrary, an increase. This finding implies that it is not the time period per se that is relevant, but the state of the pandemic development in a given country at a certain point in time. Whereas, the pandemic was reaching another peak in Eastern Europe and the Baltic States in summer 2021 (Hale et al., 2021), there were relatively low infection rates and increasing vaccination rates in most of Western Europe, possibly lowering the need and enthusiasm for social support that was common in Western Europe at the beginning of the COVID-19 pandemic.

Hypothesis 2 (*being older, male, less educated and having a migration background are associated with lower provision of instrumental help during the COVID-19 pandemic, while living in urban areas, having a paid work and a high income are associated with higher provision of instrumental help*) was largely confirmed by our analyses. However, the associations between providing instrumental help on the one side and gender and employment status on the other side became insignificant in summer 2021, which can be interpreted as a slightly decreasing impact of socio-demographic and economic characteristics on instrumental help as well as less need for help in the course of the ongoing pandemic in general. Only respondents' subjective economic situation did not exhibit the expected association, possibly due to a less clear link of subjective assessments with providing instrumental help. Future analyses should therefore focus more on objective measures, such as respondents' (household) income, which could not be included here due to questionnaire differences. The finding that older respondents had a significantly lower probability of providing instrumental help since the outbreak of the pandemic shows once again the age-related dynamics of the pandemic. Overall, our analyses clearly demonstrate the need for a differentiated consideration of a wide range of individual attributes when studying behaviors of older people during the pandemic instead of treating them as a homogeneous group.

Hypothesis 3 (*knowing people exposed to COVID-19 in their own social circles is positively associated with the provision of instrumental help, while being self-exposed to COVID-19 as well as experiencing poor health in general is negatively correlated with the provision of instrumental help during the pandemic*) was also largely supported. Knowing someone infected by COVID-19 was positively associated with providing instrumental help in both survey waves in 2020 and 2021.This finding provides an optimistic view of the development of social cohesion in European countries in the course of pandemic: after more than 1 year of coping with the pandemic and its consequences for individuals as well as society as a whole older Europeans were still willing and able to support those in need. However, it also has to be noted that a worse (physical) health was clearly negatively associated with providing instrumental help to others. While this could be expected, being self-affected by the coronavirus, probably with negative consequences for respondents' own health, also had a negative effect on providing instrumental help to others in the first phase of the pandemic. Interestingly, this association turned around completely 1 year later, possibly due to increased protection against the coronavirus by a prior infection and/or vaccination.

Hypothesis 4 (*having frequent social contacts and being a receiver of instrumental help are associated with a higher provision of instrumental help during the COVID-19 pandemic*) was only partly confirmed. As expected, a high number of social contacts was clearly associated with a higher probability to provide instrumental help during the pandemic, possibly due to a combination of both a higher awareness of demands for support from others and easier possibilities to help. With regard to reciprocity, the results were not as clear-cut, indicating the importance of carefully considering age-related differences as well as changing conditions over the course of the pandemic. It thus became clear that the observation of a negative correlation between receiving and giving instrumental help was only true for respondents aged 65 years and older. It seems plausible that these respondents receiving help were in a more vulnerable position due to the pandemic and hence were probably not able to provide help to others vice versa. For younger respondents between 50 and 64 years a positive correlation was found, partly supporting previous pre-pandemic research. However, this correlation was much more pronounced in the second SHARE Corona Survey in 2021, again pointing out the very specific situation in the beginning of the pandemic. In this respect, our study can add important insights regarding relevant factors that affect the interplay between receiving and providing help.

Finally, Hypothesis 5 (*more provision of instrumental help during the COVID-19 pandemic in countries with stricter pandemic-related policies and measures*) was partly supported. In the first phase of the pandemic, stricter measures were associated with more provision of instrumental help but only up to a certain degree. Very strict measures at the upper bound of the stringency index again reduced the provision of help to some extent. One year later, the continued control measures did not exhibit a significant effect on the provision of instrumental help anymore. Therefore, the cross-national differences in providing instrumental help by older people in Europe cannot be explained only by the pandemic-related policies and measures. Other macro-factors should be taken into consideration as well. Future research could look, for example, at the role of welfare systems during the pandemic. In this respect, previous pre-pandemic research has demonstrated that country-specific patterns of intergenerational solidarity are associated with welfare systems (e.g., Künemund and Vogel, 2006; Silverstein et al., 2020). It could be assumed that a lower level of social support is the result of well-functioning social policies in a specific country. However, previous pre-pandemic research has demonstrated that efficient social policies and generous welfare services rather encourage provision of informal assistance to family members (Motel-Klingebiel et al., 2005). Whether this holds true in the times of crisis, such as the COVID-19 pandemic, is a question for future research. Further, the support of older family members is seen as mixed responsibility of the family and the state (Daatland and Lowenstein, 2005). Cultural norms including filial obligations could also play a role for the intergenerational exchange during the pandemic. A study by Katz et al. (2003), for example, demonstrates that the differences in preferences for certain patterns of intergenerational solidarity across Europe are larger between countries than between different age groups.

There are several limitations to our analyses. First, SHARE is a representative cross-national survey of respondents aged 50 years and older. Although, a large fraction of the SHARE respondents is still in good health and has a sociable, active life and/or even is part of the working force (the “occupationally active”), it has to be considered that our sample might underrepresent the actual degree of provided instrumental help in Europe. Moreover, the specific age group of our sample has to be considered when drawing generalized conclusions based on our results. Second, we did not include data on the exchange of instrumental help before the pandemic in our study. Further analyses could strongly benefit from such inclusion to get a more comprehensive picture of the development in providing help to others. However, the questions in the regular SHARE waves were not directly comparable to the questions in the SHARE Corona Surveys and we were restricted to focus on data collected exclusively during the pandemic. A further restriction to the data were the lack of measures regarding composition and intensity of provided instrumental help and how these differed with regard to pre-pandemic times. Finally, our study focusses primarily on the supply side of instrumental help and the determinants of providing help as we were primarily interested in better understanding how and to what degree the COVID-19 pandemic and its accompanying epidemiological control measures affected the provision of help to others outside the own household (the help provided in the multigenerational households was not included in the analyses). Further research should also look more closely at the demand side and determinants of receiving help (for example the correlation between health status, living alone and reciprocity of the intergenerational exchange during the pandemic; see, e.g., Bertogg and Koos, 2022 for Germany). Regarding reciprocity, it could be argued that the sequence of receiving and providing help is of relevance. Based on the data in SHARE, it was, however, not possible to distinguish what came first, providing or receiving help, and whether providing instrumental help was a reaction of receiving help or not. Future research should therefore think carefully about study designs that allow disentangling the sequence of receiving and providing help, while simultaneously considering age-group-related dynamics over time.

In our paper, we mainly looked at the provision of instrumental help by older generations from the perspective of intergenerational solidarity. This becomes apparent especially when describing the changing flow patterns to different groups or recipients. Provision of instrumental help can be, however, also seen as a contribution to social cohesion in general. In the section of the paper where we looked at the determinants of provided instrumental help, engaging in “giving behavior” is analyzed in general as a contribution to social cohesion and not as providing help to a specific generation. In terms of the social cohesion debate, our findings support the optimistic view of an increasing solidarity especially in the beginning of the COVID-19 pandemic. But also 1 year later into the pandemic, the provision of instrumental help by older people was still regular or even increased in the European countries in which the pandemic was reaching a new peak. The exchange of instrumental help is driven by needs and resources as well as by public discourse and social policies. The decrease in providing instrumental help by persons aged 50+ in Western Europe in summer 2021 as compared to summer 2020, can be interpreted as “going back to normal” and less need for this type of informal help due to the pandemic “downtime” and widespread vaccination rather than a general decreasing solidarity in the society. However, to confirm this assumption, further research comparing the pre-pandemic and post-pandemic levels of help provision is needed.

Despite these limitations, our study provides a cross-national overview of how the provision of instrumental help by older generations has changed across Europe in the course of the COVID-19 pandemic and which factors were crucial for the provision of instrumental help during the pandemic with regard to the 50+ population. The findings of our study emphasize the dynamic nature of intergenerational solidarity: the usual patterns of flow are prone to rapid changes in times of crises. The likelihood and ability to provide assistance to others depend on a number of different individual and contextual factors that were analyzed above. The balance of costs, burdens and benefits of intergenerational exchange are being constantly (re)negotiated by involved actors in times of limited resources and restricted possibilities to offer help. Against this background, our findings provide new insights to the growing comparative research literature on intergenerational solidarity during the COVID-19 pandemic.

## Data availability statement

Publicly available datasets were analyzed in this study. This data can be found at: http://www.share-project.org/data-documentation/share-data-releases.html. All data used in our study are available free of charge to all scientific users world-wide after individual registration (http://www.share-project.org/data-access/user-registration.html). Each wave and each release is assigned a persistent DOI. In our manuscript we use data from SHARE Waves 1, 2, 3, 4, 5, 6, 7, 8 and 9 (DOIs: 10.6103/SHARE.w1.800, 10.6103/SHARE.w2.800, 10.6103/SHARE.w3800, 10.6103/SHARE.w4.800, 10.6103/SHARE.w5.800, 10.6103/SHARE.w6.800, 10.6103/SHARE.w7.800, 10.6103/SHARE.w8.800, 10.6103/SHARE.w8ca.800, 10.6103/SHARE.w9ca.800, 10.6103/SHARE.w8caintd.800, 10.6103/SHARE.w9caintd.800) that are fully available without restrictions.

## Ethics statement

The studies involving human participants were reviewed and approved by the Ethics Committee of the University of Mannheim (Waves 1 to 4). Wave 4 of SHARE and the continuation of the project were reviewed and approved by the Ethics Council of the Max Planck Society. For more details please see: http://www.shareproject.org/fileadmin/pdf_documentation/MPG_Ethics_Council_SHARE_overall_approval_29.05.2020__en_.pdf. Written informed consent for participation was not required for this study in accordance with the national legislation and the institutional requirements.

## Author contributions

All authors listed have made a substantial, direct, and intellectual contribution to the work and approved it for publication.

## Funding

The SHARE data collection has been funded by the European Commission, DG RTD through FP5 (QLK6-CT-2001-00360), FP6 (SHARE-I3: RII-CT-2006-062193, COMPARE: CIT5-CT-2005-028857, SHARELIFE: CIT4-CT-2006-028812), FP7 (SHARE-PREP: GA N°211909, SHARE-LEAP: GA N°227822, SHARE M4: GA N°261982, DASISH: GA N°283646), and Horizon 2020 (SHARE-DEV3: GA N°676536, SHARE-COHESION: GA N°870628, SERISS: GA N°654221, SSHOC: GA N°823782, SHARE-COVID19: GA N°101015924) and by DG Employment, Social Affairs and Inclusion through VS 2015/0195, VS 2016/0135, VS 2018/0285, VS 2019/0332, and VS 2020/0313. Additional funding from the German Ministry of Education and Research, the Max Planck Society for the Advancement of Science, the U.S. National Institute on Aging (U01_AG09740-13S2, P01_AG005842, P01_AG08291, P30_AG12815, R21_AG025169, Y1-AG-4553-01, IAG_BSR06-11, OGHA_04-064, HHSN271201300071C, RAG052527A) and from various national funding sources is gratefully acknowledged (see www.share-project.org).

## Conflict of interest

The authors declare that the research was conducted in the absence of any commercial or financial relationships that could be construed as a potential conflict of interest.

## Publisher's note

All claims expressed in this article are solely those of the authors and do not necessarily represent those of their affiliated organizations, or those of the publisher, the editors and the reviewers. Any product that may be evaluated in this article, or claim that may be made by its manufacturer, is not guaranteed or endorsed by the publisher.

## Footnotes

1 More details on this can be found here: https://digigov.univie.ac.at/solidarity-in-times-of-a-pandemic-solpan/.

2 A more objective measure, such as respondents' (household) income, could not be used due to differences in the way the income question was asked in the first and the second SHARE Corona Survey.

3 We ran a multilevel model with country as the level-two identifier as sensitivity check and used a different operationalization of the stringency index varying only between countries. The results did not deviate substantially from the results presented here. Further, we checked for deviations with regard to the different addressees of instrumental help. However, we only found minor differences in point estimates, which do not add new information compared to the used operationalization that sums up the different recipients of instrumental help.
