# Supplementary material for: The impact of the COVID-19 pandemic on the provision of instrumental help by older people across Europe
Source: Front Sociol. 2022 Nov 9;7:1007107. doi: 10.3389/fsoc.2022.1007107 (PMC9682231; doi:10.3389/fsoc.2022.1007107)
Supplement: Supplementary file 1 [file Table_1.DOCX]

**Supplementary Material**

Table A1: Percent of respondents providing help to others outside their own household since the outbreak of the pandemic, separated by age groups

|  | SCS1 (2020) | SCS2 (2021) |
| --- | --- | --- |
| Age (50-64 years) | 29.0 | 42.3 |
|  | [27.2; 30.8] | [39.1; 45.5] |
| N | 15,690 | 11,916 |
| Age (65+) | 12.6 | 22.4 |
|  | [12.0; 13.2] | [21.6; 23.2] |
| N | 31,805 | 35,579 |

Data: SHARE Wave 8 COVID-19 Survey 1 and SHARE Wave 9 COVID-19 Survey 2, Release version: 8.0.0 (n=47,495, respectively; weighted) with 95% confidence intervals in brackets.
