# Supplementary material for: The impact of the COVID-19 pandemic on the provision of instrumental help by older people across Europe
Source: Front Sociol. 2022 Nov 9;7:1007107. doi: 10.3389/fsoc.2022.1007107 (PMC9682231; doi:10.3389/fsoc.2022.1007107)
Supplement: Supplementary file 2 [file Table_2.docx]

**Supplementary Material**

Table A2: Multivariate logistic regression coefficients of respondent and country predictors on providing instrumental help to others

|  | SCS1 (2020) | SCS2 (2021) | Difference of coefficients (p-value) |
| --- | --- | --- | --- |
| *Socio-demographic & economic characteristics* |  |  |  |
| Age (50-64 years) | Reference category | | |
| Age (65-79) | -0.268^***^ | -0.298^***^ | 0.596 |
|  | (-7.61) | (-6.78) |  |
| Age (80+) | -0.458^***^ | -0.500^***^ | 0.973 |
|  | (-11.11) | (-11.95) |  |
| Gender (Male) | -0.078^*^ | -0.020 | 0.299 |
|  | (-2.23) | (-0.48) |  |
| Level of education (primary) | -0.155^***^ | -0.197^***^ | 0.537 |
|  | (-3.32) | (-3.62) |  |
| Born abroad | -0.060 | -0.090^*^ | 0.602 |
|  | (-1.70) | (-2.04) |  |
| Urban area of living | 0.098^**^ | 0.072 | 0.646 |
|  | (2.66) | (1.79) |  |
| Employed or self-employed | 0.120^**^ | -0.042 | 0.021^*^ |
|  | (2.76) | (-0.75) |  |
| Make ends meet (fairly) easily | -0.032 | 0.065 | 0.078 |
|  | (-0.82) | (1.67) |  |
| *Health-related characteristics* |  |  |  |
| Social exposure | 0.114^***^ | 0.098^*^ | 0.403 |
|  | (3.42) | (2.37) |  |
| Self-exposure | -0.077^*^ | 0.058 | 0.003^**^ |
|  | (-2.41) | (1.79) |  |
| Poor/fair self-rated health | -0.162^***^ | -0.192^***^ | 0.770 |
|  | (-4.14) | (-5.32) |  |
| *Behavioral characteristics* |  |  |  |
| Frequency of face-to-face contacts | 0.334^***^ | 0.261^***^ | 0.234 |
|  | (9.50) | (6.40) |  |
| *Reciprocity of instrumental help* |  |  |  |
| Help received from others (recipient <65 years) | 0.023 | 0.133^**^ | 0.222 |
|  | (0.54) | (3.10) |  |
| Help received from others (recipient 65+) | -0.081^*^ | -0.119^**^ | 0.935 |
|  | (-2.00) | (-2.88) |  |
| *Epidemiological control measures* |  |  |  |
| Stringency index | 0.084^*^ | -0.031 | 0.211 |
|  | (2.07) | (-0.78) |  |
| Stringency index X Stringency index | -0.094^*^ | 0.035 | 0.145 |
|  | (-2.25) | (0.87) |  |
| Constant | -1.604^***^ | 0.237 | - |
|  | (-4.00) | (0.32) |  |
| N | 42,918 | 42,918 | 85,836 |
| Adjusted R² | 0.108 | 0.096 | - |

Data: SHARE Wave 8 COVID-19 Survey 1 and SHARE Wave 9 COVID-19 Survey 2, Release version: 8.0.0 (weighted).
Entries are logistic regression coefficients with t-statistics in parentheses.
Significance level: *: p<.05, **: p<.01, ***: p<.001.
